# Management of Acetabular Fractures with Total Hip Replacement: A Narrative Literature Review

**DOI:** 10.3390/jpm15070282

**Published:** 2025-07-01

**Authors:** Domenico Tigani, Luigigiuseppe Lamattina, Andrea Assenza, Giuseppe Melucci, Alex Pizzo, Cesare Donadono

**Affiliations:** 1 Department of Orthopedic Surgery, Ospedale Maggiore C.A. Pizzardi, Largo B. Nigrisoli 2, 40133 Bologna, Italy; domenico.tigani@ausl.bologna.it (D.T.); luigigiuseppe.lamattina@ausl.bologna.it (L.L.); alex.pizzo@ausl.bologna.it (A.P.); 2 Department of Orthopedic Surgery, Ospedale Antonio Cardarelli di Campobasso, Contrada Tappino, 86100 Campobasso, Italy

**Keywords:** acetabular fracture, Total Hip Arthroplasty, dual mobility cup

## Abstract

Open reduction and internal fixation (ORIF) is widely regarded as the primary treatment for acetabular fractures, but limitations arise in complex cases, leading to non-anatomical reductions and increased risk of post-traumatic osteoarthritis. Given the high incidence of secondary arthritis (12–57%) following ORIF, total hip arthroplasty (THA) is often necessitated, particularly in scenarios unsuitable for ORIF, such as extensive comminution or combined femoral head and neck fractures. The surgical landscape has shifted from a traditional “fix or replace” to a more integrated “fix and replace” approach, especially beneficial in managing elderly patients with osteoporotic bones. THA is applied across various timelines, including acute (0–3 weeks), delayed (3 weeks to 3 months), and late (beyond 3 months), each presenting distinct challenges and requiring specific strategies to optimize outcomes. The importance of precise bone defect classifications and the role of dual mobility cups in reducing dislocation risks are highlighted, alongside the use of modern surgical and fixation techniques to improve stability and patient outcomes. Enhanced recovery protocols and meticulous postoperative management are critical to addressing complications, such as infections and hardware interference, tailoring treatment approaches to each patient’s needs, and advancing care for complex acetabular fractures.

## 1. Introduction

**Figure 1:**
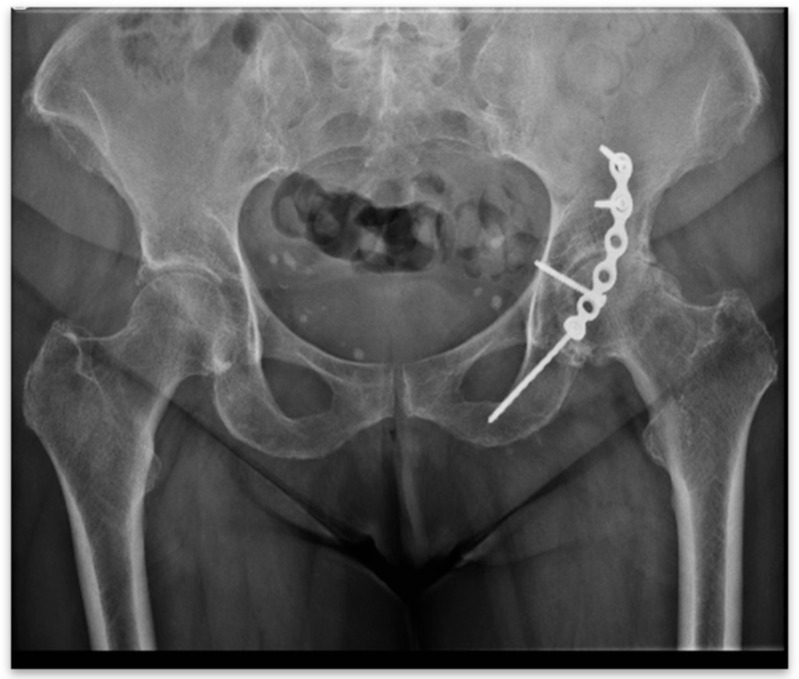
Post-traumatic arthritis.

Open reduction followed by internal fixation (ORIF) is widely considered the treatment of choice for the majority of patients affected by acetabular fractures [1,2,3]. At the same time, in cases of failure, total hip arthroplasty (THA) became the solution for secondary hip arthritis following these lesions. Restoration of congruency of the joint plays an important role in treatment outcome in the case of ORIF; residual non-anatomical reduction may lead to early surgical failure and post-traumatic osteoarthritis (Figure 1) [3,4,5,6]. A study of the literature notes a high incidence of secondary arthritis of up to 13 to 44% [7,8].

Primary total hip arthroplasty, often combined with internal fixation, can be an effective treatment for hip arthritis or in the presence of specific conditions, such as combined femoral head and/or neck fractures where satisfactory outcomes cannot be achieved with internal fixation; extensive comminution in osteoporotic bone; difficulty achieving anatomical reduction, leading to less predictable results with open reduction and internal fixation (ORIF); superior anteromedial dome impaction; and comminuted posterior wall fractures [9]. These challenges are particularly pronounced in elderly patients due to poor bone quality and the need for early mobilization. Instability remains a significant complication in both early and late total hip arthroplasty, with dislocation rates of up to 15.2% reported [10,11]. Dual mobility cups, which have been demonstrated as a valid choice to manage THA instability and to reduce the revision rate for dislocation, are preferred these days when treating acetabular fractures by THA. This article aims to analyze the indications, advantages, and limitations of primary total hip arthroplasty in the treatment of acetabular fractures while also serving as a practical guide for young surgeons approaching these complex clinical situations.

## 2. Indications and Surgical Options

The main indications for THA in the case of acetabular fractures were recently clearly exposed by Deepak Gautam et al. [12], who introduced the concept of the 4Es, as shown in Table 1.

When managing a THA for an acetabular fracture, the most important aspect is determining how much time has passed since the trauma occurred. An acute acetabular fracture, which occurs within 3 weeks, has the potential for bone healing, which makes fragments more likely to heal even with THA. Nonetheless, in these fracture types, having a stable acetabular component is crucial in reducing the likelihood of needing revisions [13,14]. The bone bed may not be adequate to amalgamate the contemporary acetabular shell designs, and an additional fixation may often be required to provide primary stability [12]. The current literature supports moving from “fix or replace” to “fix and replace” when a poor outcome is anticipated. This is particularly true for elderly patients, where various injury mechanisms and often osteoporotic bones create unique fracture patterns, usually affecting the anterior column along with posterior hemi-transverse and associated column injuries [14]. In such cases, achieving pain relief, early weight bearing, and a return to independent daily activities is crucial. From a technical point of view in the acute setting, the use of the patient’s femoral head as a structural graft, along with accurate osteosynthesis for fractures involving the posterior column, is crucial in achieving joint stability [15]. Initially, combining fixation and arthroplasty led to high complication rates, especially concerning hip instability and fixation failure [13,16,17].

With improvements in surgical techniques, more recent reports have suggested improved outcomes.

Despite the generally favorable long-term outcomes reported, specific fracture characteristics have been consistently identified as predictors of poor results. In a large cohort of over 800 patients, Matta and Tannast developed a nomogram to estimate the likelihood of requiring THA within two years after primary ORIF based on the presence of certain risk factors [18]. Building on this, Clarke-Jenssen suggested that when multiple risk factors were present simultaneously, namely, age over 60, damage to the femoral head, and acetabular impaction, the conversion rate to THA reached 100% at three years [19]. As a result, surgeons are becoming increasingly confident in anticipating which patients will eventually need THA, relying on variables such as patient age, fracture pattern, and the feasibility of achieving an anatomic initial reduction.

In clinical practice, two distinct patient profiles are becoming apparent: older individuals who typically sustain associated fractures from low-energy falls and younger patients who experience high-energy trauma with more varied fracture patterns. With a growing elderly population, the former group now represents the majority of cases presenting with negative prognostic indicators [16]. Historically, patients over 60 accounted for around 11–18% of acetabular fractures [20,21], but recent studies have reported a sharp increase in incidence among the elderly, with rates as high as 64% [22,23,24]. When managed with ORIF, the proportion of these patients eventually requiring THA for post-traumatic arthritis has been reported to range between 22 and 64% at three-year follow-up [25,26,27].

Performing THA early as part of the initial treatment is believed to offer the advantage of early mobilization, especially in elderly patients, while potentially avoiding the need for future major surgery [25,28]. This rationale has supported the growing use of acute THA in well-selected patients and has led to the publication of numerous case series and studies assessing its outcomes [25,29,30,31].

**Figure 2:**
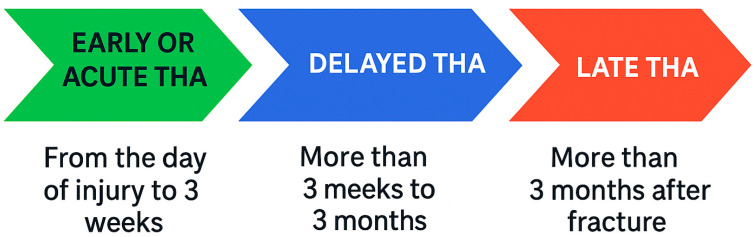
Timeline showing the three potential timings for THA in acetabular fractures.

The healing potential of a chronic fracture is variable and is considered as fibrous nonunion, thereby warranting a stable construct that bridges the fracture [32]. In terms of when to perform THA for a fractured acetabulum, THA can be considered at three time periods [33] (Figure 2).

In all cases that were previously operated on, the initial step is to rule out infection. Surgical site infections affect 5% to 7% of patients with operative fixation of acetabular fractures, with risks heightened by factors like prolonged surgery time, increased blood loss, and other complications [34]. Reviewing previous operation records for these risk elements is advisable. Elevated ESR and CRP levels necessitate hip aspiration. Other methods for infection detection are also recommended [35,36].

In cases of manifest infection, it is better to perform THA in stages. Two-stage THA consists of an initial phase involving extensive surgical debridement and removal of any existing hardware, followed by targeted antibiotic therapy and, subsequently, a second phase with the implantation of the total hip arthroplasty [35,37,38].

It is crucial to thoroughly assess the status of the sciatic nerve post-trauma or surgery, due to potential complications from previous injuries or the risk of new injuries. Documentation of any existing hardware is essential, and although routine removal is not generally recommended unless there is an infection, precise planning with a CT scan is crucial to identify any hardware that may interfere with the procedure.

Regarding the issue of instability, dislocation is also minimized using larger femoral heads and dual-mobility cups (DMCs).

## 3. Classification of Bone Defects

For years, there has not been any distinct classification to evaluate the bone defect in cases of previous acetabular fractures. The American Academy of Orthopedic Surgeons (AAOS) [39] and Paprosky classifications [40] have been widely used, even for the evaluation of acetabular fracture bone defects. However, these classifications were mainly to detect the bone loss observed in cases of arthroplasty failure. The pattern of bone defect in revision scenarios is different, characterized by osteolysis and cup migration. More recently, Marmor et al. [34] proposed a CT classification for acute acetabular fractures of geriatric patients based on mapping the stable articular surface, which helped surgeons specify the pelvic bone corridor for stable cup fixation (Table 2).

The authors classified fractures based on the available stable articular surface and intact bone corridors for acetabular cup fixation. They found that the dome is the most common stable articular surface, followed by the posterior one; the sciatic buttress corridor was available in all fracture patterns, while the gluteal pillar corridor was the second most frequently available area for secured screw fixation.

A new X-ray classification was proposed to evaluate bone defects in previously managed or neglected acetabular fractures. According to this classification, based on the acetabular fracture classification of Letournel and Judet [1], acetabular defects result from primary traumatic displacements of the acetabular wall or columns that are completely reduced, partially reduced, or not at all reduced [41]. The radiographic landmarks in the anteroposterior view of the pelvis are the (1) posterior wall, (2) ilioischial line, (3) iliopectineal line, and (4) position of the femoral head/direction of migration of the femoral head. The acetabular bone defects were graded into five types (Table 3).

## 4. Surgical Technique

The surgical approach and reconstructive procedures will necessarily be dependent on the different scenarios and the type of bone defects encountered. The surgical approach is based on the fracture location and comminution; the Kocher–Langenbeck approach is the most commonly used approach in this context. Some authors have recommended the anterior approach when there is an associated anterior column fracture [42]. In such cases, our strategy also involves utilizing a posterior approach with a cage to bridge both the defect and the fracture.

### 4.1. Delayed THA, Neglected, or Conservatively Treated Acetabular Fractures

An acetabular fracture is often classified as neglected if it has not been addressed within three weeks. Reconstructive surgery is typically recommended for neglected acetabular fractures when there is a misalignment between the femoral head and the acetabular cup. This principle also applies to acetabular fractures that have failed conservative treatment. In such instances, open reduction and internal fixation (ORIF) may not be appropriate because of potential complications like avascular necrosis of the femoral head and challenges in achieving accurate realignment of the fracture fragments.

Consequently, most of these fractures are usually managed with total hip replacement. Generally, medical unsuitability for major pelvi-acetabular surgery or deficiencies in the referral system are fundamental reasons for the delay in definitive management [43].

**Figure 3:**
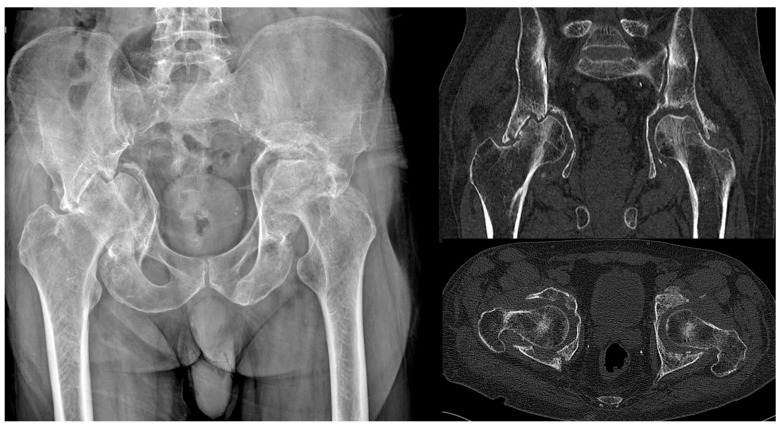
Anteroposterior pelvic radiograph and CT scans showing bilateral acetabular impaction fractures in a patient who sustained a high-energy fall. The patient refused acute treatment. The imaging demonstrates the condition three months post-injury.

**Figure 4:**
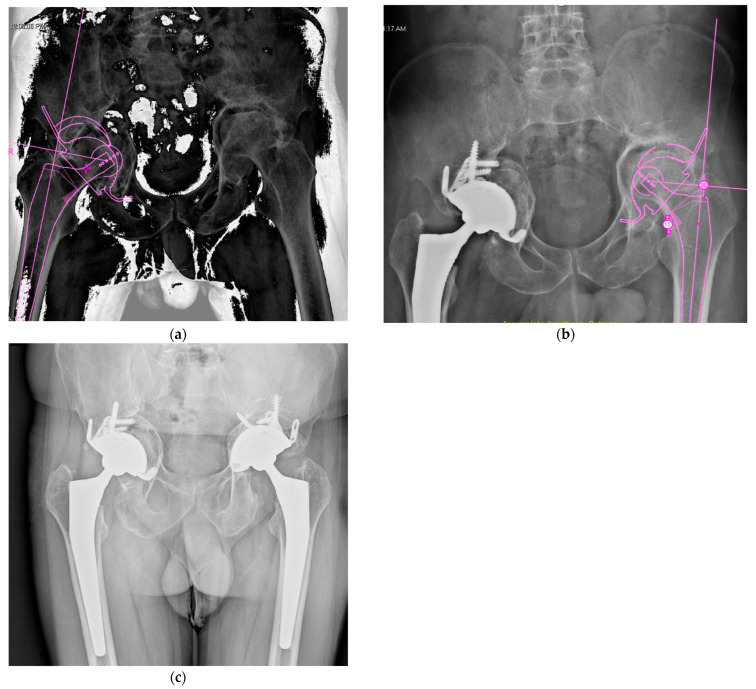
Preoperative planning images for right (**a**) and left (**b**) total hip arthroplasty showing the extent of acetabular bone loss and implant positioning strategy. (**c**) Postoperative radiograph at the 24-month follow-up.

Distorted anatomy, the presence of scar tissue and fibrosis among the bone fragments, malunion, nonunion, central hip dislocation associated with protrusio acetabuli, or pelvic discontinuity present major challenges in treating these injuries (Figure 3 and Figure 4). Additionally, numerous potential complications arise when managing aged, previously unoperated acetabular fractures, including damage to the femoral head and avascular necrosis [44,45].

#### Acetabular Exposure, Preparation, and Cup Fixation

For us, it is crucial to achieve adequate exposure of the acetabulum while preserving a posterior capsular flap for final reconstruction. Simultaneously, protection of the sciatic nerve is essential, maintaining flexion of the hip and knee throughout the procedure. The femoral head is kept apart to prepare the autograft. In type 1 defects, grafting is the preferred method for addressing protrusion deformities, which are commonly associated with an expansion of the acetabular cavity.

Although the stability of the acetabulum may not heavily depend on the posterior and anterior walls, adequately fixing a well-preserved posterior wall with screws and plates before reaming is beneficial [46]. Persistent posterior column or transverse fractures must be treated. In the case of persistent nonunion, it is possible to utilize compression plating to reduce and stabilize the pelvis to create a solid bony substrate for acetabular reconstruction.

An autograft is an appropriate method for addressing these acetabular deformities. It provides further advantages by reducing the likelihood of loosening and dislodgement, helping the capacity of healing the fracture and contributing to the restoration of the hip center [47].

A morcellized bone graft is compacted into the defect using reverse reaming. Current research indicates that patients might benefit from press-fit implants in such scenarios. Depending on the characteristics of the bone deformity and whether a three-point fixation of an acetabular cup is technically feasible within the confines of the acetabular wall, a hemispherical, cementless, multi-hole, porous metal cup is generally favored for this kind of reconstruction. A minimum of four screws should be used to fix the cup.

**Figure 5:**
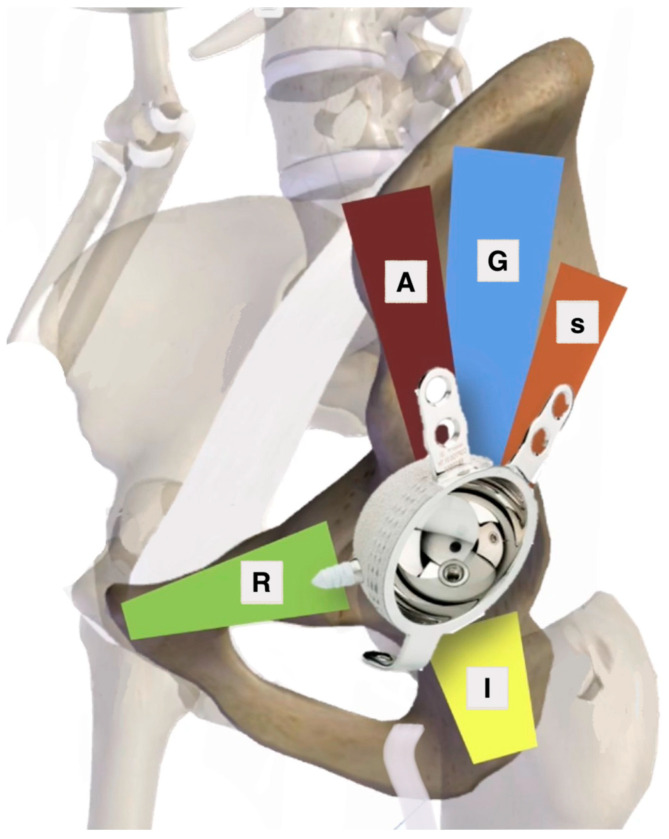
Marmor et al.’s [34] corridors for screw positioning: S = sciatic buttress corridor, G = gluteal corridor, A = anterior corridor, I = ischial corridor, and R = superior pubic ramus corridor.

According to Marmor et al.’s [34] paper, at least two corridors are available for fixation in 93% of fractures (Figure 5). Typically, the most posterior corridor, including the sciatic buttress and gluteal pillars, is readily accessible for screw fixation. To prevent cup failure during abduction, it is advised to place two short screws (ranging from 15 to 20 mm in length) inferiorly in the superior pubic ramus and ischium, commonly referred to as the “kickstand” screw.

Instability continues to be a significant concern, as a high dislocation rate has been reported in these cases [48]. Our preference is to employ a dual mobility cup, as it can significantly reduce the risk of dislocation in such cases and is currently favored in the treatment of acetabular fractures with THA. Modern designs of modular dual mobility (DM) cups facilitate the implementation of this surgical strategy by using a metal liner to convert a traditional cup into a dual mobility one [49]. Alternatively, it is still feasible to use monoblock dual mobility tripod implants, which enhance press-fit fixation in all three planes with an iliac screw and two pegs, thereby eliminating the need for an additional metal liner (Figure 3).

In more complex cases or when pelvic discontinuity is present, the use of a standard cup is not advised; instead, cage reconstruction becomes a viable alternative. The cages designed to bridge defects typically feature extra-acetabular plates placed over the ilium and distal anchorage using an obturator hook, along with a plate that is either screwed over or buried into the ischium. The cage serves to shield the graft during incorporation and remodeling and, due to its proximal and distal anchorage, contributes to the stabilization of the lesion. These constructs can accommodate an independent cemented dual mobility implant. Historically, Bursch Schneider cages and Kerboull cross-plates, spanning from the ilium to the ischium, have been extensively utilized for reconstructing severe acetabular bone deficits.

However, ring-and-cage constructs are generally not suitable for younger patients because of potential complications, such as fatigue fractures of the flanges and lack of biological fixation compared to other methods. Nevertheless, they may still be considered for the low-demand elderly population. An uncemented cup–cage system with obturator hooks and screwed flanges also remains an option, offering both standard and dual mobility configurations. These systems are constructed around a cup featuring a double surface of titanium/hydroxyapatite-coating or a trabecular titanium alloy cup for secondary osteointegration.

### 4.2. Late THA, Failure of Previously Operated Acetabular Fractures

**Figure 6:**
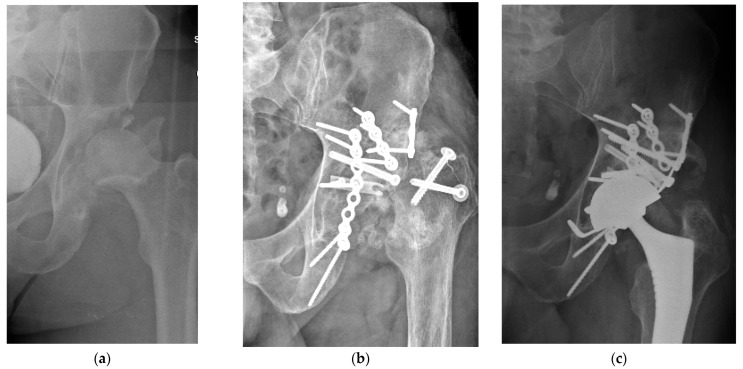
(**a**) Posterior hip dislocation with an associated posterior column fracture. (**b**) Seven-month follow-up showing femoral head osteonecrosis. (**c**) Postoperative radiograph after total hip arthroplasty.

When post-traumatic arthritis and femoral head necrosis are present, total hip arthroplasty (THA) serves as a rational salvage procedure (Figure 6). The complexity of subsequent THAs is increased because of retained internal fixation implants, scar tissue, and residual defects in the acetabular bone. Therefore, rigorous preoperative planning is of paramount importance. Careful clinical preoperative examinations should determine previous surgical accesses, limitations in movement, potential limb length discrepancies, and the presence of heterotopic ossifications. It is also crucial to assess the type and location of hardware, acetabular bone remodeling, including bone defects, zones of necrosis, and pseudoarthrosis.

From a general perspective, hardware removal is rarely required for acetabular cup positioning, as screws are typically placed away from the articular surface. Nonetheless, the potential role of retained hardware as a source of infection remains a topic of ongoing debate [37].

The use of two-stage total hip arthroplasty (THA), combined with a course of antibiotic therapy between the two procedures in patients with elevated infection markers, has been proposed; however, its definitive efficacy remains unproven [50,51].

Contained and cavitary defects are managed by impaction grafting using autologous bone from the femoral head. When the autograft is insufficient or unavailable due to extensive femoral head necrosis, trabecular metal augments or buttresses may be employed to reconstruct the defect. The use of dual mobility implants remains advantageous even in cases of osteosynthesis failure, as it contributes to a reduced incidence of dislocation. In the presence of severe chronic acetabular bone defects or pelvic discontinuity, where achieving stable initial acetabular fixation is challenging, major reconstructive procedures in conjunction with total hip arthroplasty may be required. In such complex scenarios, our preferred approach involves cage reconstruction with a cemented cup or, preferably, a cup–cage construct.

**Figure 7:**
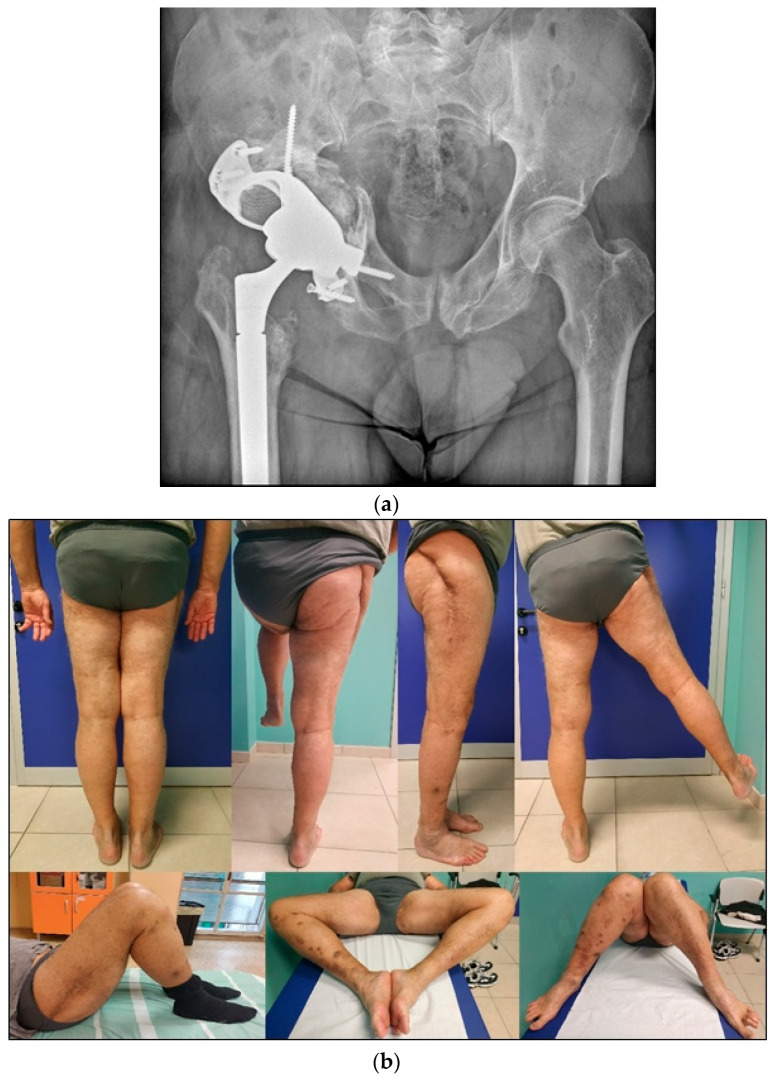
(**a**) Custom-made triflange implant. (**b**) Clinical photographs of a patient showing a complete range of motion with no pain during hip mobilization.

Finally, custom triflange acetabular components (CTACs) have been proposed as a treatment strategy for the reconstruction of extensive acetabular defects (Figure 7). These monoblock, patient-specific implants are designed to precisely fit the defect and can also provide stabilization in cases of pelvic discontinuity. Custom-made acetabular implants represent a reliable option for managing severe bone loss, enabling restoration of the center of rotation, with reported survival rates exceeding 90%. Durand-Hill et al. [52] reported encouraging accuracy in the placement of custom 3D-printed titanium components in patients with major acetabular defects, with no cases showing a discrepancy greater than 10 mm between the planned and achieved COR in the lateromedial plane.

### 4.3. Acute THA in Acetabular Fractures

The initial approach to managing acetabular fractures with immediate total hip arthroplasty (THA) began in the late 1990s. This early method involved primary stabilization using cables, followed by THA performed within the same surgical session [42].

In 1998, Mouhsine et al. [53] modified the technique by introducing the Dall–Miles cable and a single-side tensioner, which creates a figure-eight configuration around the acetabulum.

Currently, the criteria for promptly addressing an acetabular fracture with THA involve elderly patients who exhibit extensive intra-articular comminution, impaction of the acetabular dome, a displaced–impacted femoral neck fracture, severe osteopenia or osteoporosis, or pre-existing osteoarthritis. Modern plate fixation techniques and new acetabular cup designs provide improved treatment options. Primary THA, when necessary, can be performed using a posterior approach for fractures accessible in this way. An anterior approach can also be used for THA if both fracture fixation and THA are performed from the front. This distinction reflects the evolution of surgical techniques in the treatment of acetabular fractures [54].

**Figure 8:**
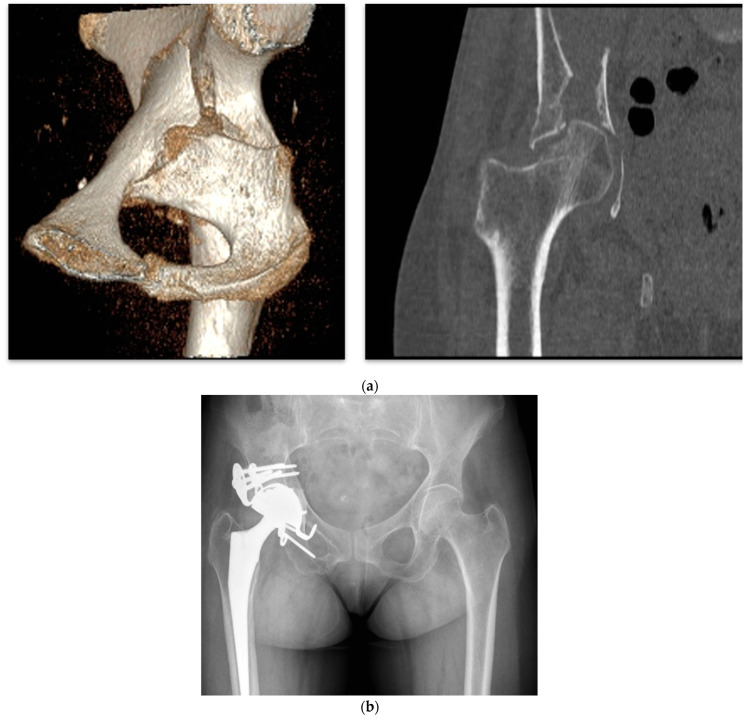
(**a**) Three-dimensional reconstruction after trauma in an elderly patient. (**b**) Acute treatment with Kerboull cross-plate fixation with a cemented cup.

In selected cases, particularly anterior wall fractures, which represent the most common fracture type, especially following low-energy trauma [55], the use of the Kerboull acetabular reinforcement device in combination with a dual mobility cup may be beneficial in low-demand patients (Figure 8). In such scenarios, the need for additional plate fixation is infrequent.

Anterior wall fractures in the elderly often present a distinct injury pattern, including a separate quadrilateral plate fragment, roof impaction in anterior column fractures with medialization of the femoral head, and comminution with marginal impaction in posterior wall or column fractures [20].

The Kocher–Langenbeck approach is typically employed to access the hip. After reaching the hip joint and performing a capsulotomy, a femoral neck osteotomy is carried out, and the head is preserved to be morcellized.

The acetabular cavity is thoroughly exposed after the removal of all loose cartilaginous fragments.

Distally, the fibrotic tissue and the transverse acetabular ligament are preserved to ensure the hook of the KP remains in contact with the distal edge of the acetabulum. If the lower margin of the acetabulum is fractured, a graft from the femoral head is placed in the hook. This autologous cancellous graft from the head is crucial for successful bone grafting, requiring a precise technique to shape the bone graft to fit the defect and pressing it into the cavity using reverse reaming.

To achieve a correct 45-degree inclination of the KP, the hook should be positioned parallel to the floor. Assi et al. [56] suggest using a small bulk graft between the iliac bone and the upper palette to facilitate positioning. Care must be taken to ensure that the horizontal flanges do not protrude outside the reconstructed bone cavity. The palette is then secured with screws before cementing a cemented dual mobility cup, downsized by one or two sizes relative to the KP.

Alternatives to the Kerboull plate (KP) include acetabular roof reinforcement devices, such as the Müller or Ganz rings, as well as anti-protrusion cages. The Müller or Ganz ring is particularly effective in fractures of the medial lamina and anterior wall. Its application requires an intact inferior acetabular rim to properly anchor the hook at the incisura acetabuli.

The Burch–Schneider anti-protrusion cage is indicated for cases involving major bone defects. However, its use demands adequate surgical exposure and experience, as screw fixation must be achieved in areas with good bone stock. The primary advantages of rings and cages include restoration of the hip center and the provision of uniform load distribution to the bone grafts, thereby promoting bone remodeling and graft incorporation into host bone [57]. The main limitation, however, is the potential for cage fracture or loosening due to the absence of biological fixation.

In cases involving posterior wall or transverse fractures, which represent the majority, surgical management typically includes compression plating to reduce and stabilize the pelvis, thereby providing a solid bony foundation for acetabular reconstruction. This is commonly performed in conjunction with simultaneous THA using a dual mobility cup. Circumferential reaming of the acetabulum is essential in all cases. Care must be taken to avoid over-reaming medially and to preserve the bleeding surface of the stable host bone.

**Figure 9:**
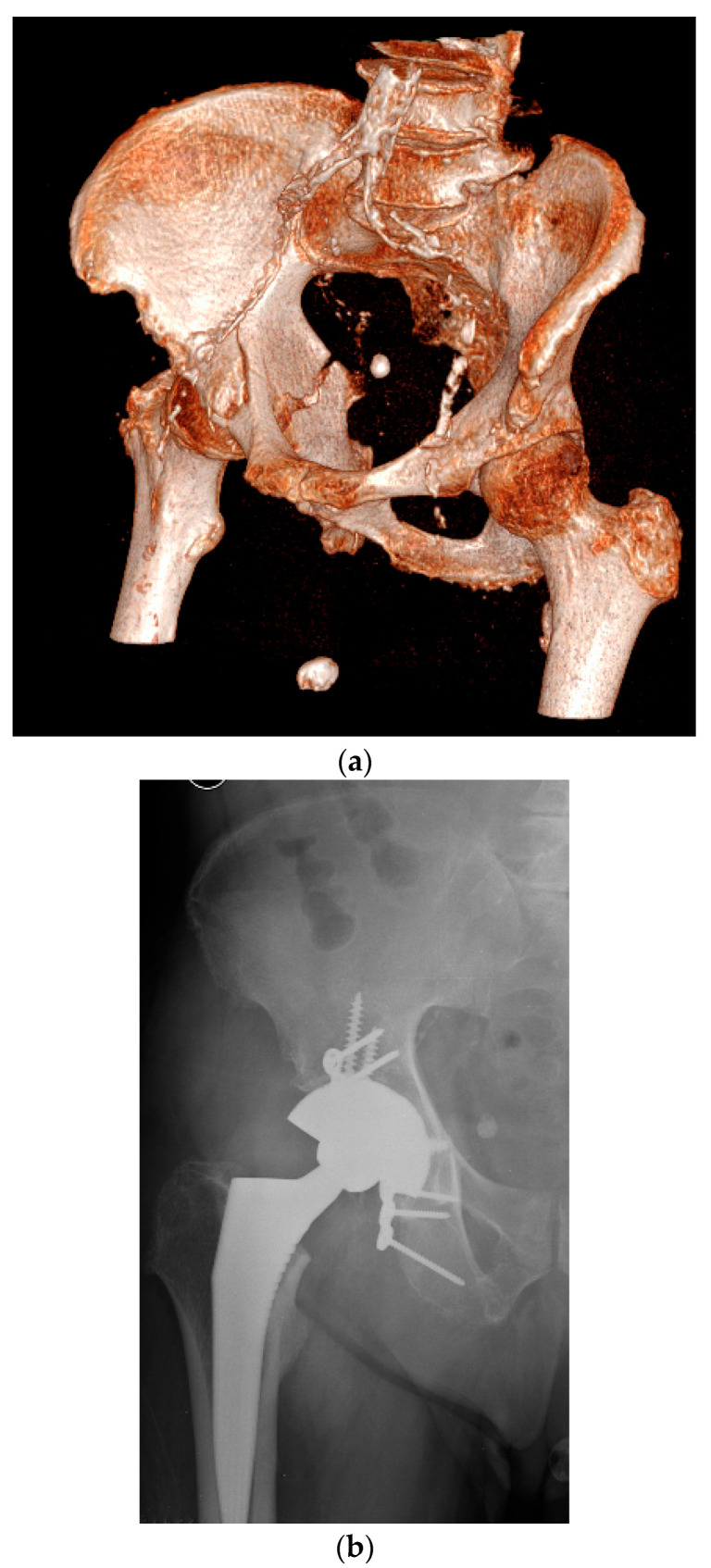
(**a**) Three-dimensional CT reconstruction. (**b**) Postoperative radiograph with standard multi-hole acetabular cup and posterior plate fixation.

Following this, the morcellized autograft harvested from the osteotomized femoral head is impacted into the defect using reverse reaming. The diameter of the final reamer dictates the outer diameter of the definitive acetabular cup. When an adequate press-fit is achievable, a cementless, porous-coated hemispherical metal acetabular implant is preferred. These implants are suitable for contained defects or defects that can be made contained, especially when sufficient rim fixation and multiple screw options are available (Figure 9).

The successful integration of these advanced surgical techniques ensures enhanced stability, reduced dislocation risks, and better overall outcomes for patients, marking a crucial advancement in the treatment of complex acetabular injuries.

## 5. Conclusions

In conclusion, total hip arthroplasty has become a crucial technique for both primary and salvage interventions in acetabular fractures, particularly useful in managing complications like osteoporosis, severe comminution, or existing arthritis. The integration of dual mobility cups has particularly improved outcomes by reducing dislocation rates and the need for subsequent revisions.

Enhancements in surgical strategies and imaging technologies have focused on preserving bone integrity, restoring proper alignment, and ensuring implant longevity through precise preoperative planning. Furthermore, the development of bone defect classifications and the strategic use of autografts and modular implants emphasize personalized surgical treatments tailored to each patient’s specific needs.

Overall, the field continues to evolve, aiming to improve recovery times, reduce complications, and enhance patient mobility. As research progresses, these approaches will likely be refined further, promising better outcomes for patients with complex acetabular fractures.

## Figures and Tables

**Table 1:** Main indications for THA in acetabular fractures according to the 4E concept described by Deepak Gautam et al. [12].

I	**E**stablished post-traumatic arthritis or avascular necrosis of the femoral head following fixation or conservative treatment.				
II	**E**xisting hip arthritis or associated fracture of the head and/or neck of the femur not amenable to a satisfactory outcome with fixation.				
III	**E**lderly patient with comminuted acetabular fractures and osteoporotic bone.				
IV	**E**xpected undesirable outcome at an early follow-up period after fixation.				
(a) Associated severe articular cartilage injury and marginal impaction of the acetabulum.		(b) Persistently subluxated head or neglected fracture dislocation with risk of avascular necrosis of the femoral head.		(c) Thin and compromised posterior wall or column with risk of fixation failure.	

**Table 2:** Classification system for acetabular fractures based on articular surface and bone stock described by Marmor et al. [34].

1. Zone of Articular Surface Fully or Partially Connected to Stable Bone	2. Bone Stock Available for Screw Fixation
D—Dome zone only P—Posterior zone only A—Anterior zone only DP—Dome and posterior DA—Dome and anterior DAP—All zones	R—Superior ramus pubic corridor A—Anterior corridor G—Gluteal pillar corridor S—Sciatic buttress corridor I—Ischium corridor

**Table 3:** Sen et al.’s [41] classification based on radiographic landmarks in an X-ray ap view of the pelvis with bilateral hips.

New Classification Type	Posterior Wall	Illioischial Line	Illiopectineal Line	Femoral Head Position/Migration
Type 1 (circumferential widening)	Present	Undisplaced	Undisplaced	Minimal
Type 2 (posterior wall)	Absent/displaced	Undisplaced	Undisplaced	Posterior superior/superolateral/ dislocated
Type 3 (posterior column)				
3A (malunion or fibrous union)	Present	Displaced but not broken	Undisplaced	Superomedial
3B (frank nonunion)	Present	Displaced and broken	Undisplaced	Superomedial
Type 4 (transverse)				
4A malunion or fibrous nonunion	Present	Displaced but not broken	Displaced but not broken	Superomedial
4B frank nonunion (pelvic discontinuity)	Present	Displaced and broken	Displaced and broken	Superomedial
Type 5 (anterior column)	Present	Undisplaced	Displaced	Medial

## Data Availability

The articles cited in this paper are available on PubMed^®^, UpToDate^®^, and Cochrane^®^.
